# Targeting cell surface GRP78-CD44v interaction suppresses cell migration in triple-negative breast cancer cells

**DOI:** 10.1038/s41598-025-33441-5

**Published:** 2025-12-21

**Authors:** Chun-Chih Tseng, Pu Zhang, Mari B. Ishak Gabra, Mei Kong, Amy S. Lee

**Affiliations:** 1 Department of Biochemistry and Molecular Medicine, University of Southern California, 1441 Eastlake Avenue, Los Angeles, 90089 CA USA; 2 USC Norris Comprehensive Cancer Center, University of Southern California, 1441 Eastlake Avenue, Los Angeles, 90089 CA USA; 3 Present Address: Department of Developmental Neurobiology, St. Jude Children’s Research Hospital, Memphis, 38105 TN USA; 4 Department of Molecular Microbiology and Immunology, University of Southern California, 1441 Eastlake Avenue, Los Angeles, 90089 CA USA; 5 Present Address: Department of Biology, University of North Carolina at Chapel Hill, Chapel Hill, 27599 NC USA; 6 Department of Molecular Biology and Biochemistry, School of Biological Sciences, University of California, Irvine, Irvine, 92697 CA USA

**Keywords:** GRP78, CD44, Src, Triple-negative breast cancer, TNBC, MDA-MB-231, Cancer, Cell biology

## Abstract

Triple-negative breast cancer (TNBC) is characterized by the absence of estrogen receptor (ER), progesterone receptor (PR), and HER-2 amplification, rendering it unresponsive to endocrine and HER2-targeted therapies. GRP78 (78 kDa glucose-regulated protein), a key endoplasmic reticulum (ER)-resident chaperone involved in protein folding and stress response, has been observed atypically localized on the cell surface of various cancer and stressed cell types, where it engages in non-canonical signaling and cellular functions. Cell surface GRP78 (csGRP78) is preferentially expressed in malignant cells relative to normal tissue, making it an attractive therapeutic target. In this study, we report that over 70% of MDA-MB-231 TNBC cells express csGRP78. Interestingly, MDA-MB-231 cells predominantly exhibit a distinct unipolar morphology, with csGRP78 prominently co-localizing with the variant isoform of CD44 (CD44v, containing variable exon 3) at the anterior region of the cell. Co-localizations of csGRP78 and CD44v were also observed in MDA-MB-231 tumor xenografts, supporting its relevance *in vivo*. Importantly, targeting csGRP78 with the monoclonal antibody 76-E6 downregulated CD44v expression, inhibited Src kinase signaling, disrupted cell morphology, and suppressed cell motility. We further mapped the epitope of GRP78 targeted by 76-E6. Together, our findings identify csGRP78 as a functional regulator of cell morphology and migration at least in part via a csGRP78-CD44v axis and underscore its potential as a therapeutic target in TNBC.

**Supplementary Information:**

The online version contains supplementary material available at 10.1038/s41598-025-33441-5.

## Introduction

Triple-negative breast cancer (TNBC) predominantly affects women under 40 years old, accounting for approximately 15–20% of breast cancer cases^1^. TNBC typically presents with a basal B type molecular profile and is characterized by early metastasis and relapse, in contrast to the luminal subtypes of breast cancer^2^. While hormone receptor-positive and HER2/Neu-amplified breast cancers have effective molecular-targeted therapies, treatment options for TNBC are mainly limited to chemotherapy and radiotherapy. Recently, immunotherapy, which modulates immune cell reactivity and enhances cancer cell-targeting, has become an increasingly important strategy for TNBC treatment. Despite initial treatment responses, recurrence remains a major cause of poor outcomes in TNBC patients^3–5^. Therefore, there is a critical need for new molecular targets for TNBC therapy.

The 78 kDa glucose-regulated protein (GRP78) is overexpressed on both total and cell surface levels in tamoxifen-resistant breast cancer and castration-resistant prostate cancer cells^6^. In TNBC patients, GRP78 expression is inversely correlated with overall survival and disease-free survival^7^. However, the specific roles of csGRP78 in TNBC cells are still largely unexplored. Recent studies have revealed novel functions of atypically localized GRP78, beyond its classical role as a chaperone in the endoplasmic reticulum (ER), in various cancers^8–12^. We previously demonstrated that csGRP78 interacts with CD44v in tamoxifen-resistant MCF7 (MCF7-LR) breast cancer cells, influencing cell adhesion, spreading, CD44v membrane dynamics, and STAT3 signaling^10,11^. CD44, particularly in its variant isoforms, is linked to the stemness phenotypes of cancer cells^13,14^. Hypoxia-induced expression of variant CD44 isoforms has been observed in MDA-MB-231 and SUM-149 TNBC cells *in vitro* and MDA-MB-231 tumor xenografts *in vivo*^15^. MDA-MB-231 cells, which are primarily unipolar with distinct anterior and posterior protrusions, are highly motile compared to the more rounded MCF7-LR cells. In this study, we utilized the unique unipolarity and rapid movement of MDA-MB-231 cells to facilitate our exploration of novel functions of csGRP78.

## Results

**Fig. 1:**
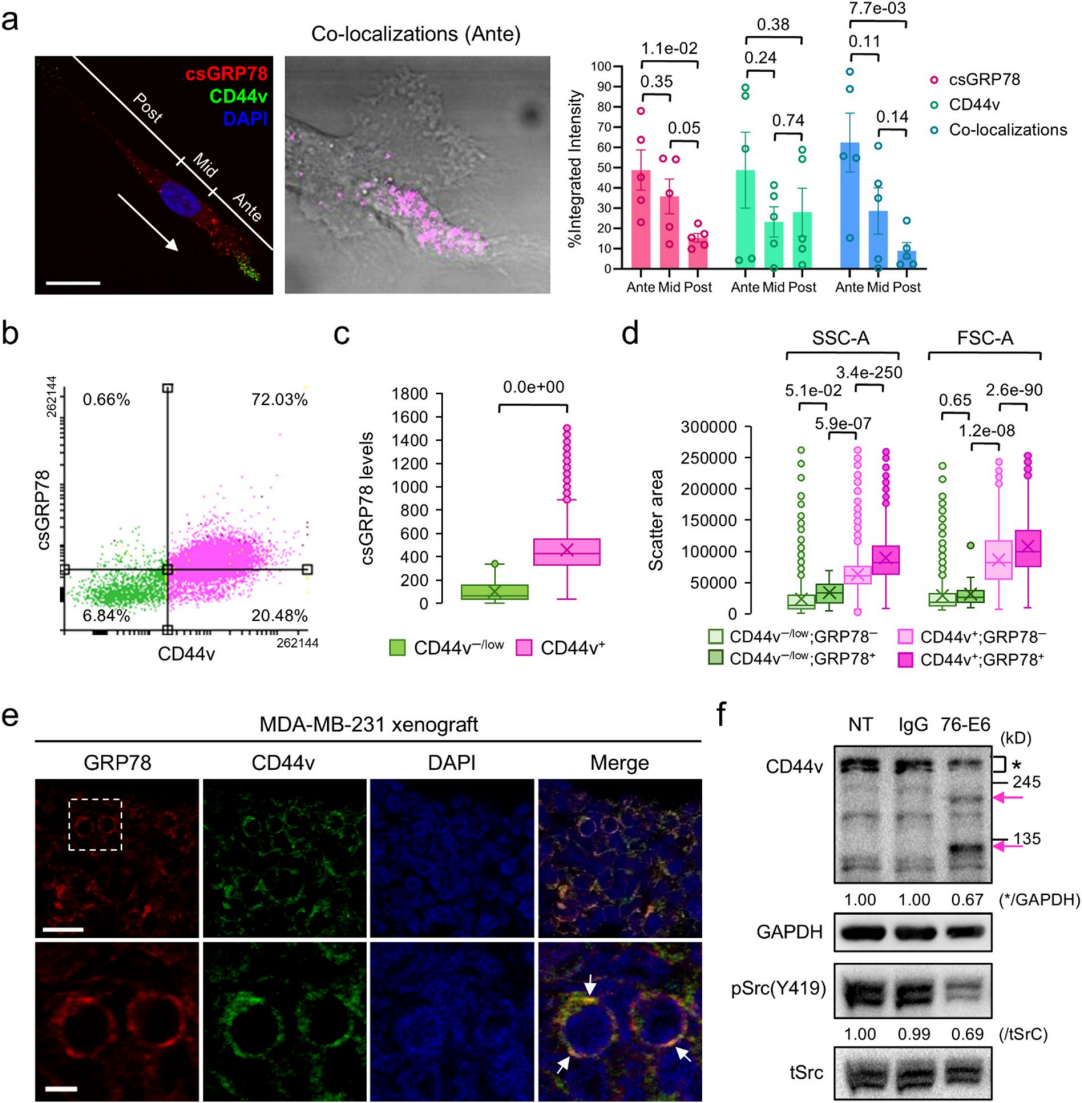
Cell surface GRP78 co-localizes with CD44v *in vitro* and *in vivo* in MDA-MB-231 breast cancer cells. (**a**) Left: Immunofluorescence and compressed z-stack confocal image of a non-permeabilized MDA-MB-231 cell showing cell surface GRP78 (red) and CD44v (green); nucleus stained with DAPI (blue). The arrow indicates the direction of cell migration. Ante, anterior; Mid, middle; Post, posterior. Scale bar, 20 μm. Middle: Co-localization of GRP78 and CD44v (magenta) at the anterior region overlaid on DIC image using FIJI-ImageJ. Right: Quantification of csGRP78, csCD44v, and co-localized signals across different regions of unipolar cells. Each dot represents one cell. Statistics: unpaired two-tailed Student’s t-test; error bars: standard error of the mean (SEM). (**b**–**d**) Flow cytometry analysis of csGRP78 and csCD44v expression on live MDA-MB-231 cells. Unpaired two-tailed Student’s t-test was used to calculate the *p*-values. (**b**) CD44v^+^ cells (magenta, *n* = 23,820); CD44v^-/low^ cells (green, *n* = 1,629). (**c**) Box-and-whisker plot of csGRP78 expression in CD44v^+^ vs. CD44v^-/low^ cells. “x” indicates the mean; dots, outliers. (**d**) Box-and-whisker plot of side scatter area (SSC-A) and forward scatter area (FSC-A) in subpopulations: CD44v^+^ GRP78^+^ (dark magenta, *n* = 3,600), CD44v^+^ GRP78^-^ (light magenta, *n* = 4,087), CD44v^-/low^GRP78^+^ (dark green, *n* = 25), CD44v^-/low^GRP78^-^ (light green, *n* = 1,663). “x” indicates the mean; dots, outliers. (**e**) Confocal images of GRP78 (red) and CD44v (green) in MDA-MB-231 tumor xenografts. Nuclei stained with DAPI (blue). The boxed region indicates the enlarged area shown in the lower panels. Arrows indicate co-localization. The thickness of the image section: 0.3 μm. Scale bars, 20 μm; 5 μm (enlarged images). (**f**) Western blot analysis of whole cell lysates from non-treated (NT) MDA-MB-231 cells and cells treated with 76-E6 antibody or control IgG for 24 h. Numbers below bands represent relative protein levels normalized to GAPDH or total Src. Magenta arrows indicate lower-molecular-weight protein forms. CD44v and GAPDH were run in the same gel while pSrc and tSrc were run in parallel on a different gel. The whole cell lysates used in the Western blot analysis were prepared from the same experiment with two biological replicates. The nitrocellulose membrane was separated into different pieces according to the molecular marker in order to blot multiple proteins. The full-length images are available in Figs. S4 and S5 of Supplementary Information 2. The raw statistical data are provided in Supplementary Information 1.

We observed csGRP78 and CD44v (CD44 containing v3 exon) exhibiting punctate expression patterns on the cell surface of MDA-MB-231 cells (Fig. 1a). While csGRP78 was predominantly expressed at the anterior and middle portions compared to the posterior area in the unipolar cells, the distribution of CD44v varies among individual cells (Fig. 1a and Supplementary Information 1). Interestingly, csGRP78 and CD44v co-localized predominantly at the anterior of the unipolar cells (Fig. 1a and Supplementary Information 1). Of the MDA-MB-231 cells, 73% expressed csGRP78 and 93% expressed CD44v on the cell surface (Fig. 1b). Additionally, 72% of the cells co-expressed both csGRP78 and CD44v (Fig. 1b). Notably, cells with higher CD44v expression also exhibited higher csGRP78 expression (Fig. 1c and Supplementary Information 1). Cell surface GRP78 and CD44v co-expressing cells showed increased cellular complexity (SSC-A) and larger cell size (FSC-A) compared to the CD44v single-positive or CD44v negative/low cells (Fig. 1d and Supplementary Information 1). In the *in vivo* model of MDA-MB-231 subcutaneous tumor xenografts, we identified punctate co-localizations of GRP78 and CD44v at the cell periphery (Fig. 1e, arrows). Importantly, treatment of the cells with the previously described^10^ anti-GRP78 antibody (76-E6) for 24 h resulted in a reduction in higher molecular weight CD44v expression (Fig. 1f, asterisk) and an increase in lower molecular weight CD44v expression (Fig. 1f, arrows in magenta). This reduction of CD44v upon 76-E6 treatment was also observed in MCF7-LR cells^10^. While CD44 lacks intrinsic kinase activity, its signaling can be mediated through direct or indirect interactions with non-receptor kinases or other cell surface receptors^16^. A previous study showed that c-Src kinase can directly bind to CD44, and that hyaluronan, a CD44 ligand, can stimulate c-Src activation and cytoskeletal activity in ovarian cancer cells^17^. In the present study, we found that 76-E6 antibody treatment in MDA-MB-231 cells resulted in reduced active Src kinase level (Fig. 1f).

To further characterize the nature of the csGRP78 and CD44v co-localizations observed by regular confocal microscopy, we first examined CD44v expression in MDA-MB-231 cells by Western blotting and found that the detected molecular sizes were consistent with our previous observations in MCF7-LR breast cancer cells and in patient-derived circulating tumor cells BRx-68 and BRx-07^10^ (Fig. S1a of Supplementary Information 2). Co-immunostaining using an antibody recognizing a shared CD44 isoform epitope (A1351) and another specific to the v3 exon (BMS144) showed that both signals overlapped, with additional CD44v puncta indicated by the arrows (Fig. S1b of Supplementary Information 2). These findings indicate that CD44 isoforms containing the v3 exon constitute the predominant CD44 species in MDA-MB-231 cells. Because we previously demonstrated direct binding of GRP78 to CD44v *in vitro*^10^, we next co-expressed FLAG-tagged GRP78 (F78) and HA-tagged CD44v (v3-v10) in 293T cells and confirmed complex formation by co-immunoprecipitation (Fig. S1c of Supplementary Information 2). We previously also showed that in super-resolution dual-color single-particle tracking, GRP78 interacts with CD44 in plasma membrane nanodomains of MCF7-LR cells^10^. To extend these findings to endogenous proteins at the cell surface, we performed LSM 880 Airyscan super-resolution imaging in non-permeabilized MDA-MB-231 cells co-stained with anti-GRP78 and anti-CD44v antibodies (Fig. S1d and e of Supplementary Information 2; and Supplementary Information 1). Image deconvolution followed by particle analysis of Airyscan datasets (40 × 40 × 170 nm voxel sampling resolution) revealed nanoscale proximity between csGRP78 and CD44v. The maximum-intensity projection illustrates the overall distribution of both signals (Fig. S1d of Supplementary Information 2), while representative Z-sections at different imaging depths (Z = 0 and Z = 2; enlarged from the boxed region in Fig. S1d of Supplementary Information 2) show puncta identified through FIJI-ImageJ particle analysis (Fig. S1e of Supplementary Information 2). Across 50 overlapping particle pairs sampled from the entire cell area, the mean centroid-to-centroid distance between csGRP78 and CD44v puncta was 113.23 ± 43.35 nm (range: 42.22–303.13 nm) (Fig. S1e of Supplementary Information 2). Given that antibody labeling introduces an expected maximal offset of approximately 30 nm per target, resulting in a total spatial separation uncertainty of ~ 60 nm, these measured distances fall well within the range compatible with co-residency in shared plasma membrane nanodomains. Although this spatial resolution cannot definitively distinguish between direct and indirect molecular interactions, the observed nanoscale proximity, when combined with biochemical evidence from co-immunoprecipitation confirming GRP78-CD44v complex formation (Fig. S1c of Supplementary Information 2) and our finding that antibody targeting of csGRP78 reduces the expression of full-length CD44v (Fig. 1f), strongly supports that csGRP78 and CD44v localize to the same plasma membrane nanodomains and that their nanoscale arrangement is functionally responsive to GRP78-directed perturbation.

**Fig. 2:**
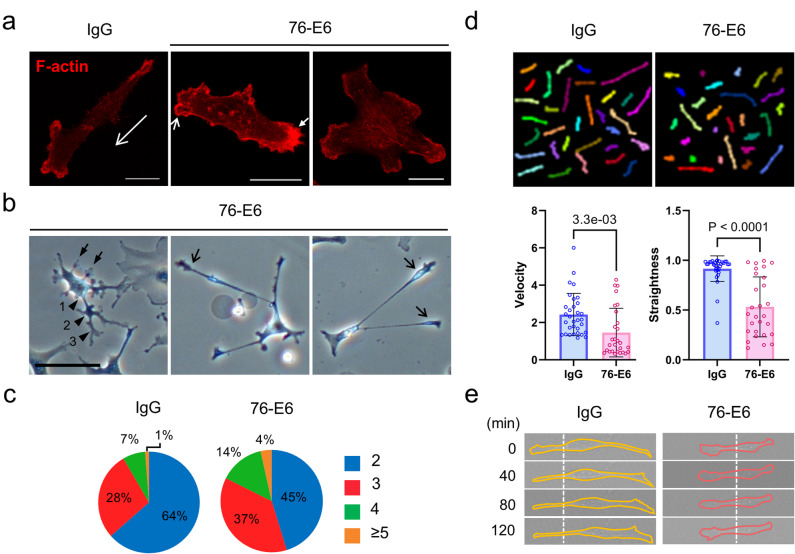
MDA-MB-231 cells treated with the anti-GRP78 antibody (76-E6) exhibit an increased number of protrusions and reduced capacity for cell migration. (**a**) Confocal micrographs showing the F-actin structures of MDA-MB-231 cells treated with 76-E6 antibody or control IgG for 24 h. F-actin was visualized through rhodamine phalloidin staining or transfected mCherry-tagged actin binding peptide. Long open arrow, direction of cell migration. Short open arrows, membrane blebs. Short solid arrow, accumulation of disorganized F-actin. Scale bars, 20 μm. (**b**) Bright-field micrographs showing the morphology of MDA-MB-231 cells treated with 76-E6 antibody for 49 h. Solid arrows, short cell protrusions. Open arrows, long cell protrusions. Arrowheads, primary (1), secondary (2), and tertiary (3) cell protrusions. Scale bar, 100 μm. (**c**) The percentage of cells displaying the indicated number of long cell protrusions after treatment with 76-E6 or control IgG for 61 h. Total number of cells analyzed in the study: 2699 (IgG); 942 (76-E6). (**d**) Upper: Superimposed tracks of control IgG (*n* = 32) and 76-E6 antibody (*n* = 28)-treated MDA-MB-231 cells during a 7-hour random migration assay. Lower: Comparisons of velocity (displacement/time) and straightness (displacement/total path length) of the cells. The unpaired two-tailed Student’s t-test was used to calculate the *p*-values; error bars represent the standard deviation (SD). The raw statistical data are provided in Supplementary Information 1. (**e**) Time-lapse DIC images of MDA-MB-231 cells during a 2-hour observation period. Solid lines, cell borders. Dotted lines, positions of the cells. min, minutes.

Furthermore, we demonstrated that MDA-MB-231 cells treated with the 76-E6 antibody for 24 h exhibited disorganized F-actin structures and membrane blebbing (Fig. 2a, solid arrow and short open arrow, respectively), similar to what we previously reported in MCF7-LR cells^10^. Consistent with the effects of 76-E6, siRNA- or shRNA-mediated down-regulation of *Grp78* (si*Grp78*) or *CD44* (sh*CD44*) recapitulated the morphological and cytoskeletal defects observed in 76-E6-treated cells (Fig. S2a and d of Supplementary Information 2, open arrows). This convergence of phenotypes indicates that the cell surface pool of GRP78 is a key functional regulator of F-actin organization in MDA-MB-231 cells. The siRNAs used in this study were previously validated^10^, and the knockdown efficiency of shCD44s was confirmed by flow cytometry (Fig. S2c of Supplementary Information 2). Surprisingly, a higher percentage of 76-E6-treated MDA-MB-231 cells (24–61 h) displayed more than two major cell protrusions, and this phenotype was not observed in the siRNA- or shRNA knockdown groups (Fig. 2a–c). These cells exhibited various protrusion phenotypes, including long protrusions (open arrows), short protrusions (solid arrows), and primary, secondary, and tertiary branches at the indicated numbered locations (Fig. 2b). Additionally, 76-E6 treatment reduced the velocity and straightness of MDA-MB-231 cells during a 7-hour recording of spontaneous cell migration, which began 24 h after treatment (Fig. 2d and Supplementary Information 1). A separate 2-hour recording revealed restricted local membrane activities without significant cell movement in 76-E6-treated cells (Fig. 2e). Consistently, *Grp78* knockdown resulted in ~ 60% reduction in directed cell migration (Fig. S2b of Supplementary Information 2), and a previous study has shown that *CD44* knockdown similarly suppresses directed cell migration in MDA-MB-231 cells^18^. Taken together, these results show that csGRP78 and CD44 are critical functional regulators of the migratory properties of MDA-MB-231 cells.

**Fig. 3:**
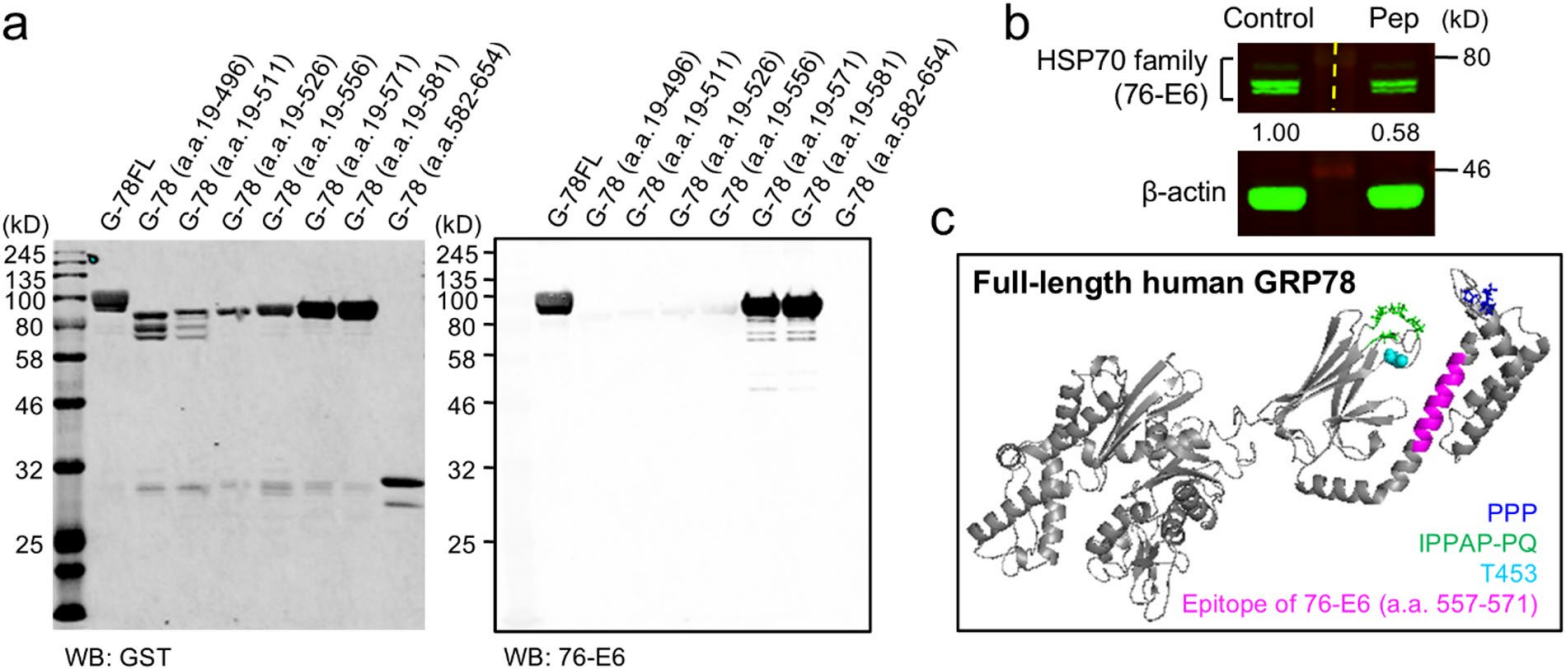
Epitope mapping of the 76-E6 antibody. (**a**) Western blot analysis of GST-tagged GRP78 full-length and deletion mutants purified from *E. coli* (BL21) using an antibody against GST (left) or GRP78 (76-E6, right). G, GST. a.a., amino acids. FL, full-length (a.a. 19–654). The experiment was run on the same gel and blot in duplicate. The full-length images are available in Fig. S8 of Supplementary Information 2. (**b**) Western blot analysis of whole cell lysate (15 µg per condition) from MDA-MB-231 cells using the equal amount of 76-E6 antibody pre-incubated with 100 µM synthetic peptide representing a.a. 557–571 of human GRP78 (Pep; ERIDTRNELESYAYS) or a control scrambled peptide sequence (TIARNSEERSYDLYE). β-actin serves as a loading control. Numbers below the GRP78 bands represent the relative levels of detected GRP78, derived from the ratio of GRP78 to β-actin. The dashed line indicates where the nitrocellulose membrane was separated for primary antibody incubation. Duplicated experiments were performed for Western blot analysis. The full-length image and the experimental procedure are available in Fig. S9 of Supplementary Information 2. (**c**) I-TASSER model and PyMOL presentation of full-length human GRP78 (a.a. 19–654), demonstrating the 3-dimensional localizations of the proline-rich sequences PPP (a.a. 640–642, in blue) and IPPAP/PQ (a.a. 487–491 and a.a. 495–496, in green), the amino acid critical for substrate binding (T453, in cyan), and the mapped epitope of the 76-E6 antibody (a.a. 557–571, in magenta).

We next aimed to explore the mechanism of action of the 76-E6 antibody by identifying its epitope. The 76-E6 antibody was originally developed by Dr. John F. Kearney at the University of Alabama, Birmingham, and the information of its epitope, located within a broad region of the human GRP78 amino acid sequence (a.a. 497–581), is publicly available. Using this information, we designed GRP78 deletion mutants and produced GST-tagged GRP78 recombinant proteins in *E. coli* (BL21). We then performed Western blot analysis using the 76-E6 antibody and found that GRP78 proteins containing amino acids 557–571 and 557–581 showed strong and similar immunoblot intensities (Fig. 3a), suggesting that the 76-E6 antibody targets amino acids 557–571 of the human GRP78 protein. In support, pre-incubation of the 76-E6 antibody with a synthetic peptide comprising a.a. 557–571 of GRP78 largely reduced the antibody’s immunoreactivity (Fig. 3b). For additional insights into the functional roles of the 76-E6 antibody, we visualized its epitope on the I-TASSER-simulated GRP78 protein structure^19–21^ (Fig. 3c). We found that the spatial location of a.a. 557–571 is in proximity to threonine 453 (T453), which is important for GRP78’s substrate binding function as demonstrated by the T453D mutant in previous studies^22,23^. Interestingly, a.a. 557–571 is also spatially close to two proline-rich regions. These include the tri-proline area (PPP; a.a. 640–642), which is critical for GRP78’s cell surface re-localization and signaling function^11^, and the IPPAP-PQ region (a.a. 487–496)^11^ with proline 495 known to facilitate substrate binding of GRP78^24^. The spatial proximity of these regions to the epitope of the 76-E6 antibody suggests that the 76-E6 antibody could potentially perturb the functions of these motifs. This warrants further investigation.

Lastly, we previously reported that the 76-E6 antibody unexpectedly recognizes both GRP78 (HSPA5) and HSP70 (HSPA1A), members of the heat shock protein 70 family. Using refined epitope information, we performed a multiple sequence alignment with Clustal Omega and found that the GRP78 proteins in both mouse and human share an identical 76-E6 epitope sequence (Fig. S3a of Supplementary Information 2, highlighted in yellow). In contrast, human HSP70 and GRP78 exhibit 53% sequence identity within the 76-E6 epitope region (Fig. S3a of Supplementary Information 2). Previous studies have shown that a subfraction of both GRP78 and HSP70 can localize to the plasma membrane. However, GRP78 predominantly exists as a peripheral membrane protein with the 76-E6 epitope accessible extracellularly, whereas HSP70 is primarily an integral membrane protein, exposing only limited regions outside the 76-E6 epitope on the cell surface^22,25^. To investigate whether HSP70 could be expressed in a peripheral form and thus be targetable by the 76-E6 antibody in MDA-MB-231 cells, we conducted immunofluorescence and confocal microscopy on non-permeabilized cells using an HSP70-specific antibody (clone C92F3A-5). No detectable surface expression of HSP70 was observed by confocal imaging (Fig. S3b of Supplementary Information 2). We next employed a more sensitive biochemical approach by analyzing purified biotinylated cell surface proteins. Western blotting revealed only a barely detectable surface pool of HSP70 in MDA-MB-231 cells compared with MCF7-LR cells (Fig. S3c of Supplementary Information 2). This minimal signal may reflect limited extracellular exposure of HSP70, its indirect association with membrane proteins in large complexes that become biotinylated, or a small fraction of dead/compromised cells with exposed intracellular HSP70. Together, the concordant imaging and biochemical results indicate that HSP70 is minimally present on the outer surface of MDA-MB-231 cells, suggesting that the functional effects of 76-E6 observed in this study are predominantly mediated through targeting csGRP78. Nonetheless, these findings do not exclude the possibility of intracellular HSP70 being targeted by internalized 76-E6, either via endosomal escape or through disruption of csGRP78-associated HSP70 complexes, if such interactions occur. These potential mechanisms warrant further investigation.

## Discussion

We previously reported that GRP78 and CD44v co-localize on the cell surface of MCF7 tamoxifen-resistant breast cancer cells and breast cancer patient-derived circulating tumor cells^10^. However, those cell types rarely exhibit distinct unipolar morphology. In contrast, MDA-MB-231 cells, which are predominantly unipolar, allowed us to observe intriguing co-localizations of csGRP78 and CD44v at the cell anterior (Fig. 1a). This suggests that GRP78 and CD44v may form complexes on the cell surface of MDA-MB-231 cells, potentially mediating cell functions related to chemotaxis, polarity, migration, and metastasis. Further investigation is needed to explore these possibilities.

The punctate expression, co-localization patterns, and measured centroid-to-centroid distances between 42.22 and 303.13 nm (mean: 113.23 ± 43.35 nm) (Fig. 1a and e; and Fig. S1d and e of Supplementary Information 2) suggest that csGRP78 and CD44v co-reside in plasma membrane nano- and/or micro-domains, which aligns with previous observations in MCF7-LR cells^10^. Importantly, treatment with the anti-GRP78 (76-E6) antibody reduced the high molecular weight CD44v expression in MDA-MB-231 cells (Fig. 1f), similar to our findings in MCF7-LR cells^10^. This indicates a functional nanodomain association between CD44 and csGRP78, and that csGRP78 contributes to maintaining CD44v plasma membrane homeostasis across different breast cancer cell types. Previous studies have shown that CD44, when associated with lipid rafts, can regulate cytoskeletal dynamics by activating Src family protein kinases^26–28^. We observed a decrease in Src activation following 76-E6 treatment, suggesting that Src may be a downstream signaling molecule in the csGRP78-CD44v axis in MDA-MB-231 cells (Fig. 1f). Interestingly, we found that STAT3 signaling could be the primary downstream pathway of csGRP78 in MCF7-LR cells^11^. These cell type-specific signaling pathways and their functional implications require further investigation.

7﻿6-E6 antibody treatment disrupted F-actin integrity in both MDA-MB-231 (Fig. 2a) and MCF7-LR cells^10^. Interestingly, only MDA-MB-231 cells treated with 76-E6 antibody showed a clear increase in protrusion numbers (Fig. 2a–c). It would be intriguing to further explore the underlying mechanisms and the identity/polarity of these protrusions. The reduced motility and migratory capacity of MDA-MB-231 cells upon 76-E6 treatment *in vitro* (Fig. 2d) is likely a combined effect of altered cell signaling, disrupted F-actin, and perturbed cell morphology.

Overall, our study highlights novel roles of cell surface GRP78 in regulating cell morphology, migration, and Src signaling in MDA-MB-231 triple-negative breast cancer cells. Further investigation is required to elucidate the underlying mechanisms and to define the specific functions of csGRP78-CD44v interactions in cancer. Importantly, the refined epitope information of the 76-E6 antibody opens up new opportunities to develop novel therapeutics targeting csGRP78-CD44v interactions. The α-helical structure at and around the a.a. 557–571 area will allow us to create various stapled peptides targeting GRP78-interacting partners specifically on the cell surface and/or in the intracellular space and design more efficient/specific reporters for high-throughput screening targeting GRP78-CD44 interactions. Anticipated advances in targeting csGRP78 and its interacting partners will usher in precision medicine applicable to a wide range of diseases beyond cancer, where csGRP78 is the functional regulator of the pathological process.

### Limitations of this study

This study broadens our understanding of csGRP78 by demonstrating its nanoscale co-residency with CD44v on the surface of MDA-MB-231 cells, showing its influence on CD44v membrane homeostasis, and revealing its impact on Src activity, cytoskeletal organization, and cell migration. Several areas, however, require additional investigation. Although super-resolution imaging allowed us to resolve csGRP78 and CD44v within shared nanodomains, this approach cannot determine whether the two proteins engage directly or through intermediary membrane components. The co-immunoprecipitation data supporting their association was generated from an overexpression system in 293T cells, leaving unanswered whether similar complexes form endogenously in TNBC cells. The observed shift in CD44v molecular species following GRP78 perturbation also remains mechanistically undefined, as the underlying biochemical events were not explored here. While our data indicate that 76-E6 primarily acts on csGRP78 in MDA-MB-231 cells, a minor contribution from HSP70, either through limited surface exposure or antibody internalization, cannot be excluded. Moreover, the downstream pathways perturbed by csGRP78 appear to differ between cell types, suggesting that the signaling architecture surrounding csGRP78 may vary across breast cancer subtypes. We also inferred nanodomain organization from imaging alone, and future work employing biochemical fractionation or raft/subdomain markers will be needed to define these membrane territories more precisely. Finally, although we confirmed csGRP78 and CD44v co-localization in tumor xenografts, the functional consequences of targeting csGRP78 *in vivo* were not assessed. Collectively, addressing these unanswered questions will help establish a deeper and more comprehensive understanding of how csGRP78 coordinates plasma membrane organization and signaling across different breast cancer contexts and will guide the development of more refined therapeutic approaches.

## Methods

### Animals

All animal experiments were conducted in accordance with protocols approved by the Institutional Animal Care and Use Committee (IACUC) at the University of California, Irvine, and followed all relevant ethical guidelines. Tumor xenografts were established in female immunodeficient mice using MDA-MB-231 cells as previously described^29^. This study is reported in accordance with the ARRIVE guidelines.

### Cell culture and antibodies

MDA-MB-231 cells were originally obtained from Dr. Shyamala Maheswaran (Harvard Medical School, MA) and cultured in Dulbecco’s modified Eagle’s medium (DMEM) supplemented with 10% fetal bovine serum, 4 mM L-glutamine, 4.5 g/L glucose, 100 IU/mL penicillin, and 100 µg/mL streptomycin. Tamoxifen-resistant MCF7 breast cancer cells were generously provided by Dr. Rachel Schiff (Baylor College of Medicine, TX) and maintained following the procedures described previously^10,11^. Only mycoplasma-negative cells were used in the study. For functional experiments, cells were treated with 50 µg/mL anti-GRP78 (76-E6) antibody or the corresponding rat IgG in antibiotic-free culture media containing 1% serum. The ABP-mcherry plasmid was constructed by replacing the EGFP sequence in the pEGFP-N2 plasmid with the LifeAct-mcherry sequence. The following primary antibodies were used: CD44v3 from Thermo Fisher Scientific (BMS144, Waltham, MA); CD44 from ABclonal (A1351, Woburn, MA); GRP78 (76-E6) from Abcam (ab25192, Cambridge, MA); GRP78 (MAb159) from Dr. Parkash S. Gill at USC (as a gift); HSP70 (C92F3A-5) from Santa Cruz Biotechnology, Inc. (sc-66048, Dallas, TX); GST tag from Santa Cruz Biotechnology, Inc. (sc-138, Dallas, TX); HA tag from Santa Cruz Biotechnology, Inc. (sc-805, Dallas, TX); FLAG M2 from Sigma-Aldrich (F1804, St. Louis, MO); GAPDH from Santa Cruz Biotechnology, Inc. (sc-32233, Dallas, TX); β-actin from Sigma-Aldrich (A5316, St. Louis, MO); Phospho-Src (Y419) from Cell Signaling Technology (2101, Danvers, MA); Src from Cell Signaling Technology (2110, Danvers, MA); and Annexin II from BD Biosciences (610068, San Jose, CA). Secondary antibodies were purchased from Santa Cruz Biotechnology, Inc. (Dallas, TX) and LI-COR Biosciences (Lincoln, NE).

### Flow cytometry analysis

Flow cytometry was performed as previously described^10^ on an LSR II (BD Biosciences), and data were analyzed using FCS Express or FlowJo v10 software. Cells were collected with a non-enzymatic cell dissociation solution (Sigma-Aldrich, C5914).

### Immunofluorescence and confocal microscopy

To detect endogenous GRP78 and CD44 containing v3 exon or HSP70 on the cell surface of MDA-MB-231 cells, cells were cultured for 48 h to subconfluence on sterile coverslips. The coverslips were sequentially coated with 50 µg/ml poly-L-lysine in ultrapure water (Sigma-Aldrich, St. Louis, MO) at room temperature (RT) for 1 h, followed by 100 µg/mL collagen I from rat tail (Corning Inc., Corning, NY) in 0.02% acetic acid at RT for 2 h. Cells were fixed in 4% paraformaldehyde (Electron Microscopy Sciences, Hatfield, PA) in Dulbecco’s phosphate-buffered saline (DPBS) at RT for 10 min and blocked with 4% bovine serum albumin (BSA) in PBS at RT for 1 h. The primary antibody against GRP78 (MAb159) was incubated with the cells at 4 °C overnight in blocking buffer, followed by staining with AlexaFluor-594 or AlexaFluor-568 secondary antibody (Thermo Scientific, Waltham, MA) at RT for 1 h. Next, the cells were treated with M.O.M. mouse Ig blocking reagent (Vector Laboratories, Burlingame, CA) at RT for 2 h to block mouse immunoglobulin from the primary mouse anti-GRP78 antibody. The cells were then incubated with the primary antibody against CD44 variable exon 3 (Thermo Scientific, Waltham, MA) or HSP70 (Santa Cruz Biotechnology, Inc., Dallas, TX) at 4 °C overnight in blocking buffer, followed by staining with AlexaFluor-488 or AlexaFluor-647 secondary antibody (Thermo Scientific, Waltham, MA) at RT for 1 h. Each step was followed by 4 washes in PBS. Coverslips were rinsed once with ultrapure water (Sigma-Aldrich, St. Louis, MO) before mounting with Vectashield anti-fade medium containing DAPI (Vector Laboratories, Burlingame, CA). For the co-staining of GRP78 and CD44v3, Z-stack images were obtained on a Zeiss LSM 510 confocal microscope equipped with a Plan-Apochromat 100x, 1.4 NA oil DIC objective lens, a Hamamatsu R6357 photomultiplier, and LSM 510 version 4.2 SP1 acquisition software (Carl Zeiss, Oberkochen, Germany). For the co-staining of GRP78 and HSP70, Z-stack images were obtained on a Leica TCS SP8 confocal microscope equipped with a 63x/1.4 NA oil immersion DIC objective and Leica Application Suite X Software (Leica Microsystems, Wetzlar, Germany).

For positive control staining of HSP70, cells cultured on coverslips were fixed with 4% paraformaldehyde at RT for 15 min, followed by permeabilization with 0.1% Triton X-100 at RT for 5 min. Cells were then blocked with 4% BSA in PBS at RT for 1 h. The primary antibody against HSP70 (Santa Cruz Biotechnology, Inc., Dallas, TX) was diluted in blocking buffer and applied to the cells, followed by overnight incubation at 4 °C. The next day, cells were incubated with an AlexaFluor-488 secondary antibody (Thermo Scientific, Waltham, MA) at RT for 1 h. Coverslips were then mounted using Vectashield anti-fade medium with DAPI (Vector Laboratories, Burlingame, CA). Z-stack images were acquired using a Leica TCS SP8 confocal microscope equipped with a 63x/1.4 NA oil immersion DIC objective and Leica Application Suite X Software (Leica Microsystems, Wetzlar, Germany).

For the detection of endogenous GRP78 and CD44 containing v3 exon in tumor xenografts, frozen sections of MDA-MB-231 tumor xenografts^29^ at the thickness of 7 μm were placed onto Superfrost Plus Micro Slide (VWR International) and stained using a similar staining protocol described above. Z-stack images were obtained on a Leica TCS SP8 confocal microscope equipped with a 63x/1.4 NA oil immersion DIC objective and Leica Application Suite X Software (Leica Microsystems, Wetzlar, Germany).

### Image analysis

Unipolar cells were categorized into three major regions of interest based on the position of the nuclei, as shown in Fig. 1a. Signal intensity was quantified using sum projections of Z-stack images in FIJI-ImageJ. Co-localized signals from individual image slices were first processed using the Image Calculator tool in FIJI-ImageJ prior to generating the sum projections.

### Super-resolution imaging and image analysis

Super-resolution imaging was conducted on a Zeiss LSM 880 AxioObserver equipped with a 32-channel GaAsP Airyscan detector and a Plan-Apochromat 63×/1.4 oil DIC M27 objective. Z-stacks were acquired in Airyscan SuperResolution mode at 16-bit depth. Quantitative analyses, including particle identification and centroid-to-centroid distance measurements, were performed in FIJI-ImageJ using the acquisition-defined pixel dimensions (40 × 40 × 170 nm in X, Y, and Z). Centroid distances were calculated from fifty overlapping GRP78-CD44v particle pairs identified across three Z-sections encompassing the full cell area.

### Purification of cell surface proteins for Western blot analysis

Experiments were performed as previously described^22^. Briefly, cells were biotinylated on ice for 30 min using 0.5 mg/ml EZ-Link Sulfo-NHS-SS-Biotin (Thermo Fisher Scientific, Waltham, MA). Excessive biotin was quenched with 100 mM glycine in cold PBS, after which cells were lysed in IP lysis buffer (Thermo Fisher Scientific, Waltham, MA). Biotin-labeled surface proteins in the resulting lysates were subsequently isolated using high-capacity NeutrAvidin agarose resin (Thermo Fisher Scientific, Waltham, MA).

### Western blotting

Cells were lysed using radioimmunoprecipitation (RIPA) lysis buffer, which contains 50 mM Tris-HCl pH 7.5, 150 mM NaCl, 0.5% sodium deoxycholate, 1% NP-40, 0.1% SDS, and a protease and phosphatase inhibitor cocktail (Roche, Indianapolis, IN). The whole cell lysates were cleared by centrifugation at 4 °C and 13,000 rpm for 15 min. Proteins were then analyzed by 10% SDS-PAGE and transferred overnight at 4 °C onto nitrocellulose membranes (Bio-Rad Laboratories, Hercules, CA). Membranes were blocked in Tris-buffered saline containing 0.05% Tween-20 (TBST) and 5% non-fat dry milk at room temperature for 1 h, followed by incubation with the primary antibody at 4 °C overnight. Membranes were washed three times with TBST and subsequently incubated with either fluorescent IRDye-labeled antibodies or horseradish peroxidase (HRP)-conjugated secondary antibodies. Fluorescent IRDye signals were detected using the Odyssey Imaging System (LI-COR Biosciences, Lincoln, NE). HRP signals were visualized with ECL chemiluminescent substrate (Thermo Fisher Scientific, Waltham, MA) and quantified using Image Lab software (Bio-Rad Laboratories, Hercules, CA).

### Plasmids and cloning

The pGEX-4T-1 bacterial expression plasmid for glutathione S-transferase (GST) tag was obtained from GE Healthcare (Chicago, IL). Full-length GST-tagged GRP78 (a.a. 19–654) and truncated GRP78 mutants (a.a. 19–496, 19–511, 19–526, 19–541, 19–556, 19–571, 19–581, and 582–654) were generated by PCR amplification of the GRP78 coding sequence from the FLAG-tagged human GRP78 (wild-type) expression plasmid^6^ and inserted in-frame into the pGEX-4T-1 expression plasmid at the BamHI and XhoI restriction sites. The following primers were used for amplification: GST-GRP78 (FL), 5’-CGCGGATCCATGGAGGAGGAGGACAAGAAGGAGGA-3’ and 5’-CCGCTCGAGCTACAACTCATCTTTTTCTGCT-3’; GST-GRP78 (a.a. 19–496), 5’-CGCGGATCCGAGGAGGAGGACAAGAAGGAGGA-3’ and 5’-CCGCTCGAGCTACTGTGGGACCCCACGAG-3’; GST-GRP78 (a.a. 19–511), 5’-CGCGGATCCGAGGAGGAGGACAAGAAGGAGGA-3’ and 5’- CCGCTCGAGCTACACTCGAAGAATACCATTCACATCTATC-3’; GST-GRP78 (a.a. 19–526), 5’-CGCGGATCCGAGGAGGAGGACAAGAAGGAGGA-3’ and 5’- CCGCTCGAGCTAGATTGTGATCTTATTTTTGTTCCCTGTACC-3’; GST-GRP78 (a.a. 19–541), 5’-CGCGGATCCGAGGAGGAGGACAAGAAGGAGGA-3’ and 5’- CCGCTCGAGCTACATCCTTTCGATTTCTTCAGGTGTC-3’; GST-GRP78 (a.a. 19–556), 5’-CGCGGATCCGAGGAGGAGGACAAGAAGGAGGA-3’ and 5’- CCGCTCGAGCTACTTGAGCTTTTTGTCTTCCTCAGC-3’; GST-GRP78 (a.a. 19–571), 5’-CGCGGATCCGAGGAGGAGGACAAGAAGGAGGA-3’ and 5’- CCGCTCGAGCTAAGAATAGGCATAGCTTTCCAACTCA-3’; GST-GRP78 (a.a. 19–581), 5’- CGCGGATCCGAGGAGGAGGACAAGAAGG-3’ and 5’- CCGCTCGAGCTACTTTTCTTTATCTCCAATCTGATTC-3’; and GST-GRP78 (a.a. 582–654), 5’- CGCGGATCCCTGGGAGGTAAACTTTCCTCTG-3’ and 5’- CCGCTCGAGCTACAACTCATCTTTTTCTGCTG-3’.

Short hairpin RNA (shRNA) targeting the *CD44* standard exon (sh*CD44*-2), as described previously^30^, or variant exon 3 (sh*CD44*v3) were cloned into the pLKO.1 vector using AgeI and EcoRI. Scrambled controls with matched nucleotide composition were generated for each shRNA. All plasmids were validated by Sanger sequencing. Primers used were: sh*CD44*-2 5’-CCGGTGTAACACCTACACCATTATCCTCGAGGATAATGGTGTAGGTGTTACATTTTTG-3’ and 5’-AATTCAAAAATGTAACA CCTACACCATTATCCTCGAGGATAATGGTGTAGGTGTTACA-3’; sh*CD44*v3 5’-CCGGCATTGATGATGATGAAGATTTCTCGAGAAATCTTCATCATCATCAATGTTTTTG-3’ and 5’-AATTCAAAAACATTGATGATGATGAAGATTTCTCGAGAAATCTTCATCA TCATCAATG-3’; shCtrl-1 5’-CCGGGCTATCATACTCCTCTAAACACTCGAGTG TTTAGAGGAGTATGATAGCTTTTTG-3’ and 5’-AATTCAAAAAGCTATCATA CTCCTCTAAACACTCGAG TGTTTAGAGGAGTATGATAGC-3’; shCtrl-2 5’-CCGGGAA TTAAGCGGATTGTATTTA CTCGAGTAAATACAATCCGCTTAATTCTTTTTG-3’ and 5’-AATTCAAAAAGAATTAAGCGGATTGTATTTACTCGAGTAAATACAATCCGCTTAATTC-3’.

### Co-immunoprecipitation assay

The FLAG-tagged full-length human GRP78 (F78) construct and the hemagglutinin (HA)-tagged CD44v3-10 (CD44v-HA) expression plasmid, both generated in the pcDNA3 vector backbone, have been described previously^11^. Subconfluent 293T cells were co-transfected with the plasmids using BioT reagent (Bioland Scientific, Paramount, CA) following the manufacturer’s protocol. Medium was refreshed approximately 16 h post-transfection, and cells were collected 48 h later for co-immunoprecipitation. Harvested cells were lysed in Pierce IP Lysis Buffer (Thermo Fisher Scientific, Waltham, MA). Lysates were precleared with Dynabeads Protein G (Thermo Fisher Scientific, Waltham, MA) and then incubated overnight at 4 °C with 1 µg mouse anti-FLAG antibody (Sigma-Aldrich, St. Louis, MO) or normal mouse IgG. Immune complexes were captured with an additional Dynabeads Protein G for 1 h at 4 °C and eluted by heating in 2x SDS sample buffer for 5 min. Samples were resolved by 10% SDS-PAGE and analyzed by Western blotting.

### Expression and purification of GST-tagged GRP78 Recombinant proteins

Plasmids encoding GST-tagged full-length GRP78 or its deletion mutants were transformed into *E. coli* (BL21). Protein expression was induced with 4 mM isopropyl-β-D-thiogalactoside (IPTG) when the optical density (OD600) of bacterial culture reached 0.5. The cultures were then incubated at 37 °C and 200 rpm for 4 h to facilitate protein expression. Cells were then lysed in TBS buffer (50 mM Tris-Cl, pH 7.5, 150 mM NaCl) supplemented with 1 mg/ml lysozyme, 1% Triton X-100, and protease/phosphatase inhibitor cocktails (Thermo Scientific, Waltham, MA). The lysate was sonicated for 4 min (20 s on, 20 s off) and subsequently centrifuged at 4 °C and 11,500 rpm for 1 h to remove cell debris. The supernatant was collected and incubated with Glutathione-Sepharose 4B beads (GE Healthcare, Chicago, IL) at 4 °C for 12 h. Recombinant GST-tagged proteins were eluted from the beads with 10 mM freshly prepared reduced glutathione (Sigma-Aldrich, St. Louis, MO) at 4 °C for 12 h. The eluted proteins were then subjected to buffer exchange to TBS using protein concentrators (Pall Corporation, Port Washington, NY). The final protein preparation in TBS, containing 15% glycerol, was snap-frozen in liquid nitrogen and stored at -80 °C for long-term storage.

### Gene knockdown

For short interfering RNA (siRNA)-mediated knockdown, cells were transfected with Lipofectamine™ RNAiMAX (Thermo Fisher Scientific, Waltham, MA) in the presence of Dharmacon siRNAs at a final concentration of 60 pM. The following previously validated^10^ sequences were used: si*Grp78*, 5′-GGAGCGCAUUGAUACUAGAdTdT-3′, and a non-targeting control (sictrl), 5′-GAGAUCGUAUAGCAACGGUdTdT-3′.

For shRNA-mediated suppression of CD44, plasmids encoding sh*CD44*-2, sh*CD44*v3, or their scrambled counterparts were introduced into MDA-MB-231 cells using the BioT transfection reagent (Bioland Scientific, Paramount, CA). As CD44-downregulated cells exhibit reduced tolerance to standard antibiotic selection^30^, a brief 24-h selection with puromycin (1 mg/mL) was applied, followed by a two-week recovery period in antibiotic-free medium. Single cell-derived clones were subsequently obtained by limiting dilution in 96-well plates (BD Biosciences, San Jose, CA), and only wells containing colonies derived from individual cells were expanded to establish stable CD44 knockdown lines.

### Scratch-wound migration assay

Cells were transfected with either non-targeting control siRNA (sictrl) or siRNA targeting *Grp78* (si*Grp78*) in triplicate and cultured for 72 h in 6-well plates until confluent. Prior to initiating the migration assay, cells were treated with mitomycin C (10 µg/mL; Roche, Indianapolis, IN) for 4 h to inhibit proliferation. A linear wound was generated using a 200 µL pipette tip, and debris was removed by gentle washing. Bright-field images were acquired at three marked positions per well immediately after scratching (0 h) and again at 24 h. Wound closure was quantified using FIJI-ImageJ.

### Cell tracking and track analysis

MDA-MB-231 cells were seeded at low density in the inner well (ø12 mm) of a collagen I-coated glass bottom dish (100 µg/mL, Ted Pella Inc., Redding, CA) 24 h prior to treatment with either 76-E6 antibody or control IgG for an additional 24 h. Time-lapse differential interference contrast (DIC) images were captured using an LSM 510 confocal microscope equipped with a 10x objective lens for the 7-hour cell tracking or a 20x objective lens for the 2-hour imaging experiment. The culture medium was overlaid with mineral oil (Sigma-Aldrich, St. Louis, MO) to prevent evaporation, and the temperature and CO_2_ levels were controlled throughout the imaging process. Images were acquired every 5 min for the 7-hour tracking experiment or every minute for the 2-hour imaging experiment. The pinhole was fully opened to reduce laser power usage and optimize the depth of the field. Cells undergoing mitosis or apoptosis were excluded from the analysis. Straightness and velocity of cell movement were quantified using the MTrackJ plugin in FIJI-ImageJ software.

### Statistics

Data are represented as mean ± standard deviation (SD) or standard error of the mean (SEM), as specified in the figure legends. Statistical analyses were performed using Microsoft Excel or GraphPad Prism version 10. Statistical significance was assessed using unpaired two-tailed Student’s t-test. The number of biological replicates and sample sizes are detailed in the respective figure legends.

#### Reporting summary

Further information on research design is available in the Nature Research Reporting Summary linked to this article.

## Supplementary Information

Below is the link to the electronic supplementary material.

Supplementary Material 1

Supplementary Material 2

## Data Availability

All data supporting the statistical analyses in this study are provided in Supplementary Information 1. Full-length Western blot images are included in Figs. S4–S10 of Supplementary Information 2. Additional data or materials are available from the corresponding authors upon reasonable request.
