# Correction: Targeting cell surface GRP78-CD44v interaction suppresses cell migration in triple-negative breast cancer cells

**DOI:** 10.1038/s41598-026-43679-2

**Published:** 2026-03-16

**Authors:** Chun-Chih Tseng, Pu Zhang, Mari B. Ishak Gabra, Mei Kong, Amy S. Lee

**Affiliations:** 1 Department of Biochemistry and Molecular Medicine, University of Southern California, 1441 Eastlake Avenue, Los Angeles, 90089 CA USA; 2 USC Norris Comprehensive Cancer Center, University of Southern California, 1441 Eastlake Avenue, Los Angeles, 90089 CA USA; 3 Present Address: Department of Developmental Neurobiology, St. Jude Children’s Research Hospital, Memphis, 38105 TN USA; 4 Department of Molecular Microbiology and Immunology, University of Southern California, 1441 Eastlake Avenue, Los Angeles, 90089 CA USA; 5 Present Address: Department of Biology, University of North Carolina at Chapel Hill, Chapel Hill, 27599 NC USA; 6 Department of Molecular Biology and Biochemistry, School of Biological Sciences, University of California, Irvine, Irvine, 92697 CA USA

Correction to: *Scientific Reports* 10.1038/s41598-025-33441-5, published online 21 December 2025

The original version of this Article contained an error in Fig. 2, where the y-axis of the bar graph is partially cropped and not fully legible. The incorrect Figure 2 along with its captions is provided below.

**Fig. 2:**
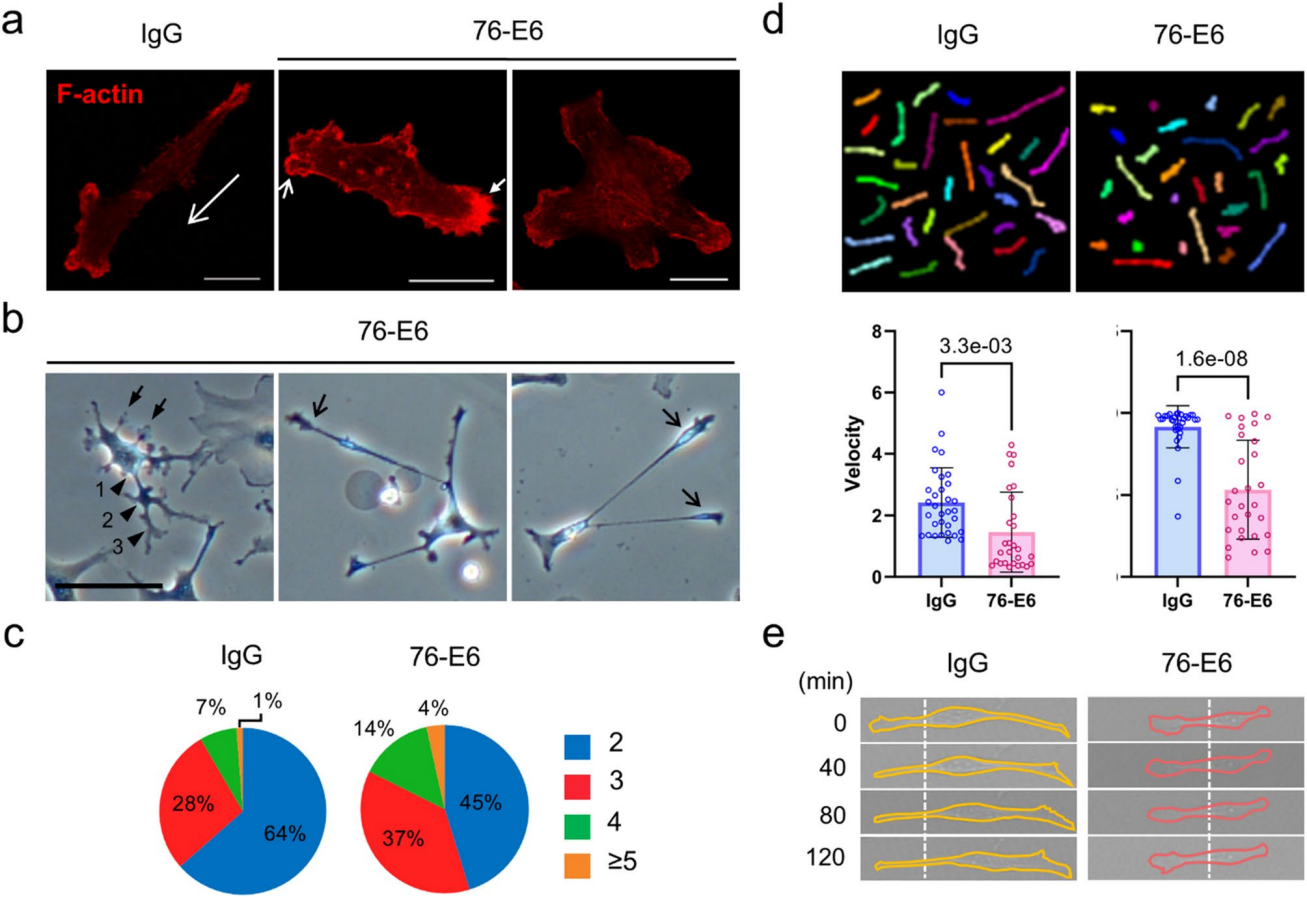
MDA-MB-231 cells treated with the anti-GRP78 antibody (76-E6) exhibit an increased number of protrusions and reduced capacity for cell migration. (**a**) Confocal micrographs showing the F-actin structures of MDA-MB-231 cells treated with 76-E6 antibody or control IgG for 24 h. F-actin was visualized through rhodamine phalloidin staining or transfected mCherry-tagged actin binding peptide. Long open arrow, direction of cell migration. Short open arrows, membrane blebs. Short solid arrow, accumulation of disorganized F-actin. Scale bars, 20 μm. (**b**) Bright-field micrographs showing the morphology of MDA-MB-231 cells treated with 76-E6 antibody for 49 h. Solid arrows, short cell protrusions. Open arrows, long cell protrusions. Arrowheads, primary (1), secondary (2), and tertiary (3) cell protrusions. Scale bar, 100 μm. (**c**) The percentage of cells displaying the indicated number of long cell protrusions after treatment with 76-E6 or control IgG for 61 h. Total number of cells analyzed in the study: 2699 (IgG); 942 (76-E6). (**d**) Upper: Superimposed tracks of control IgG (*n* = 32) and 76-E6 antibody (*n* = 28)-treated MDA-MB-231 cells during a 7-h random migration assay. Lower: Comparisons of velocity (displacement/time) and straightness (displacement/total path length) of the cells. The unpaired two-tailed Student’s t-test was used to calculate the *p*-values; error bars represent the standard deviation (SD). The raw statistical data are provided in Supplementary Information 1. (**e**) Time-lapse DIC images of MDA-MB-231 cells during a 2-h observation period. Solid lines, cell borders. Dotted lines, positions of the cells. min, minutes.

The original Article has been corrected.

