# Supplementary material for: Targeting cell surface GRP78-CD44v interaction suppresses cell migration in triple-negative breast cancer cells
Source: Sci Rep. 2025 Dec 21;16:3424. doi: 10.1038/s41598-025-33441-5 (PMC12834970; doi:10.1038/s41598-025-33441-5)
Supplement: Supplementary file 2 — Supplementary Material 2 [file 41598_2025_33441_MOESM2_ESM.pdf]

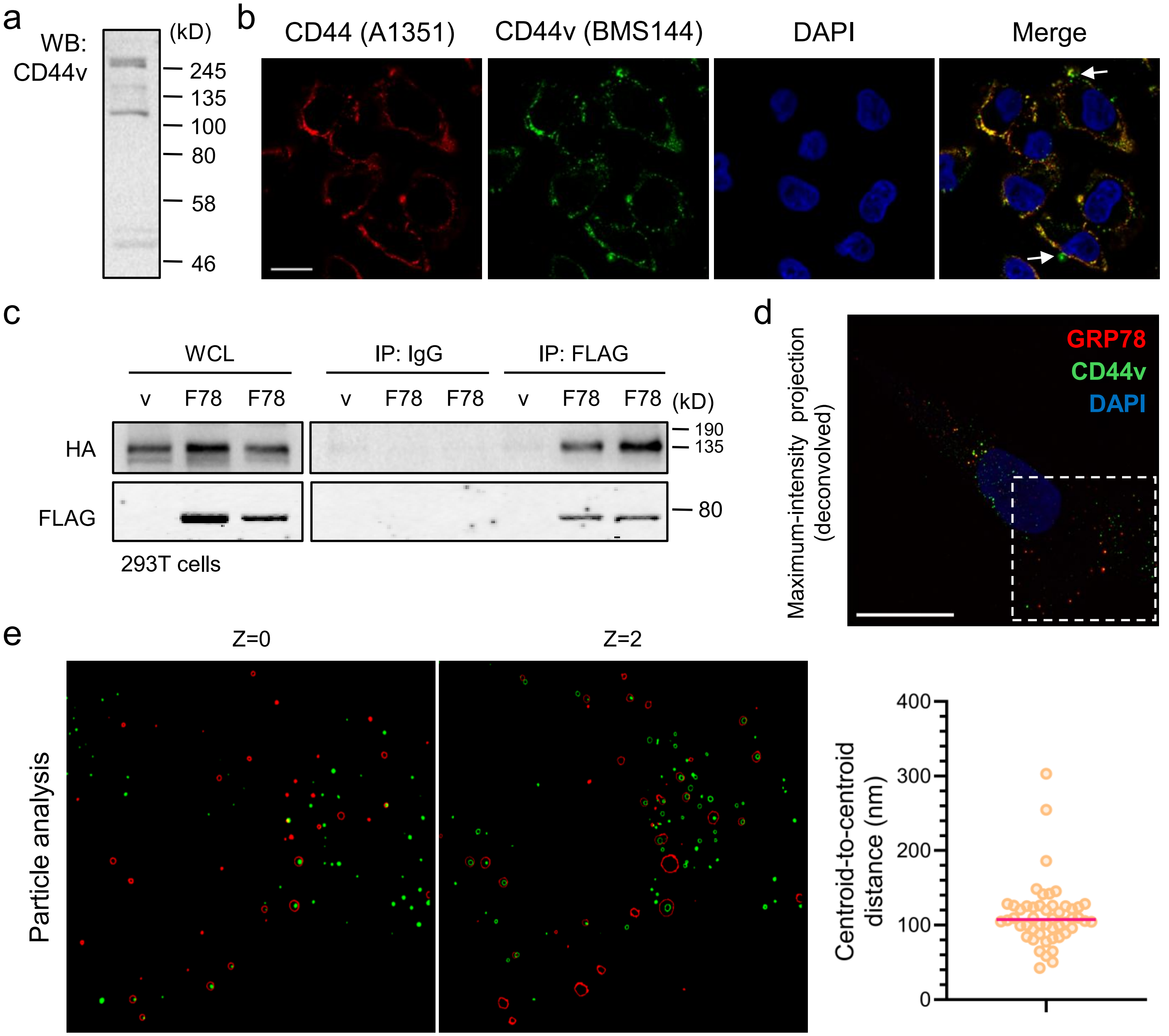

Fig. S1 The CD44 isoform containing variant exon 3 (CD44v) is the predominant isoform in MDA-MB-231 cells and co-resides with cell surface GRP78 in plasma membrane nanodomains. a Western blot analysis of MDA-MB-231 whole-cell lysates using an antibody specific for the CD44v3 exon. The full-length images are available in Fig. S6 of Supplementary Information 2. **b** Immunofluorescence and confocal images showing the surface distribution and co-localization of CD44 (A1351, red) and CD44v3 (BMS144, green) in non-permeabilized MDA-MB-231 cells. The A1351 antibody recognizes a common CD44 epitope, whereas BMS144 specifically detects the CD44v3 isoform. Arrows: CD44v3 puncta. Optical section thickness: 0.3 μm. Scale bar: 20 μm. **c** 293T cells were co-transfected with HA-tagged CD44v (exons v3–v10) and either FLAG-tagged GRP78 (F78) or the empty pCDNA3 vector (v). Cell lysates were prepared in IP lysis buffer and subjected to immunoprecipitation using an anti-FLAG antibody or isotype IgG control. FLAG-GRP78 and CD44v-HA in whole-cell lysates and IP fractions were detected by immunoblotting with anti-FLAG and anti-HA antibodies. The full-length images are available in Fig. S7 of Supplementary Information 2. **d** Super-resolution deconvolved LSM880 Airyscan confocal images showing the maximum-intensity projection of cell-surface GRP78 (red) and CD44v (green) in non-permeabilized MDA-MB-231 cells. The nucleus was stained with DAPI (blue). The boxed area is enlarged in the following panel. Scale bar: 20 μm. **e** Left: Representative images from two Z-sections showing the surface distribution of GRP78 (red) and CD44v (green) identified by particle analysis in FIJI-ImageJ. Right: Centroid-to-centroid distances at pixel-level resolution (not optical resolution) between overlapping GRP78 and CD44v particles (N = 50), quantified from Z-sections covering the full cell area. Mean ± SD = 113.23 ± 43.35 nm; the bar denotes the mean. Pixel resolutions: X=40 nm, Y=40 nm, Z=170 nm. The raw statistical data are provided in Supplementary Information 1.

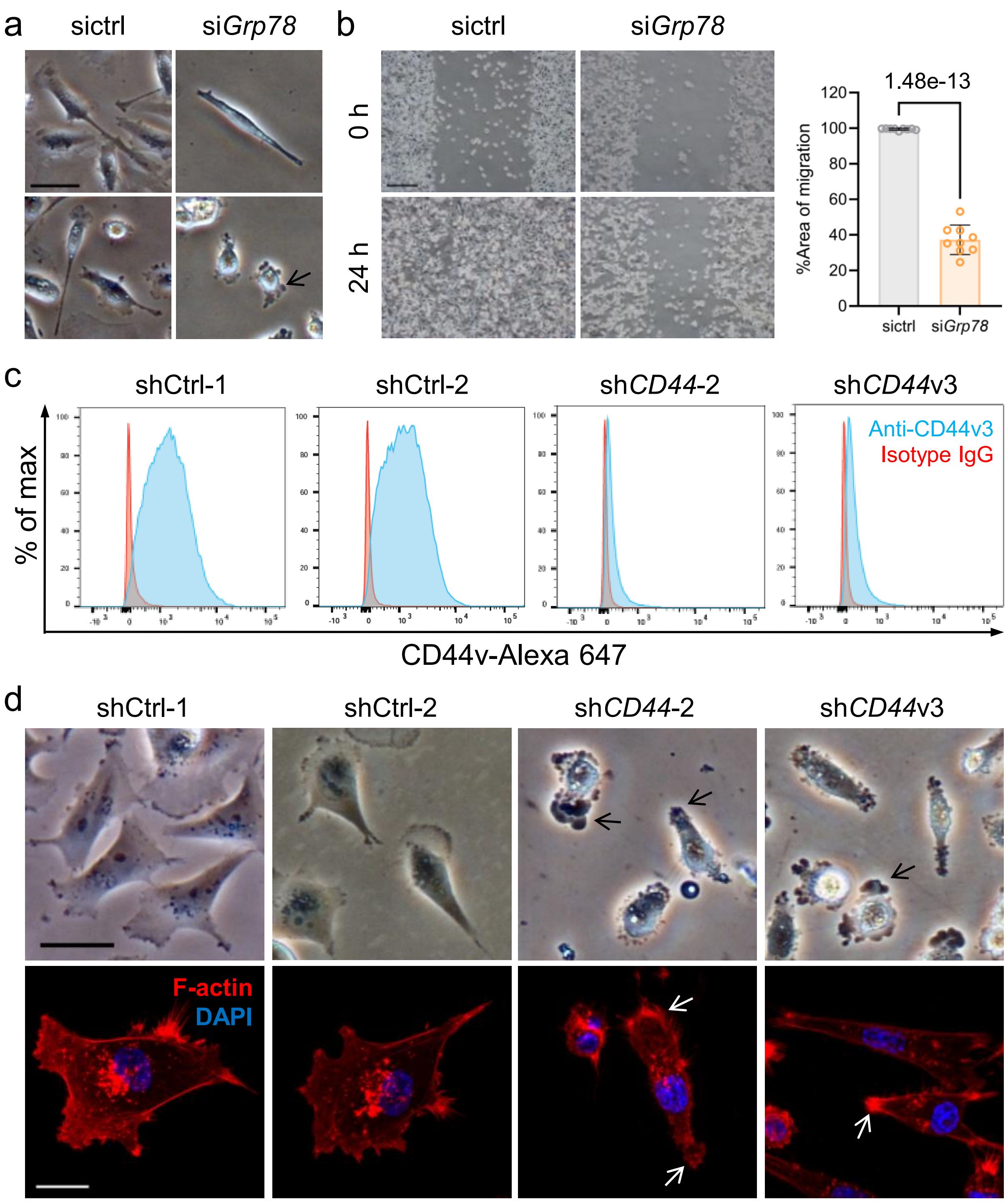

Fig. S2 Knockdown of GRP78 or CD44 alters cell morphology and disrupts cytoskeletal integrity in MDA-MB-231 cells. a Bright-field micrographs showing the morphology of cells transfected with sictrl or siRNA targeting *Grp78*. The open arrow: disorganized cytoskeletal structure and cell morphology. Scale bar, 20 μm. **b** Left: Representative bright-field images of MDA-MB-231 cells transfected with control siRNA (sictrl) or siGrp78. Cell migration was evaluated at 0 h and 24 h on a light microscopy. Right: Percent migration area was quantified in FIJI-ImageJ. Three biological replicates were analyzed per group, with three marked regions measured for each replicate. Data are shown as mean±SD, and statistical significance was assessed by Student *t* test. Scale bar: 100 μm. The raw statistical data are provided in Supplementary Information 1. **c** Flow cytometry histograms showing surface CD44v expression in stable MDA-MB-231 cell lines expressing shRNAs targeting the common CD44 sequence (sh44-2), the variant exon 3 (sh44v3), or scrambled controls (shCtrl-1 and shCtrl-2). CD44v was detected using the CD44v3-specific antibody. Cyan: anti-CD44v3; red: isotype IgG. “% of max” indicates the percentage of maximal fluorescence intensity. Approximately 30,000 cells were analyzed per sample. **d** Upper: Bright-field images of stable MDA-MB-231 cells in culture. Lower: Confocal images of F-actin organization in cells seeded on collagen I-coated coverslips. F-actin was labeled with rhodamine phalloidin (red). Nuclei were stained with DAPI (blue). Open arrows: disorganized F-actin structures and cell morphology. Scale bars: 20 μm.

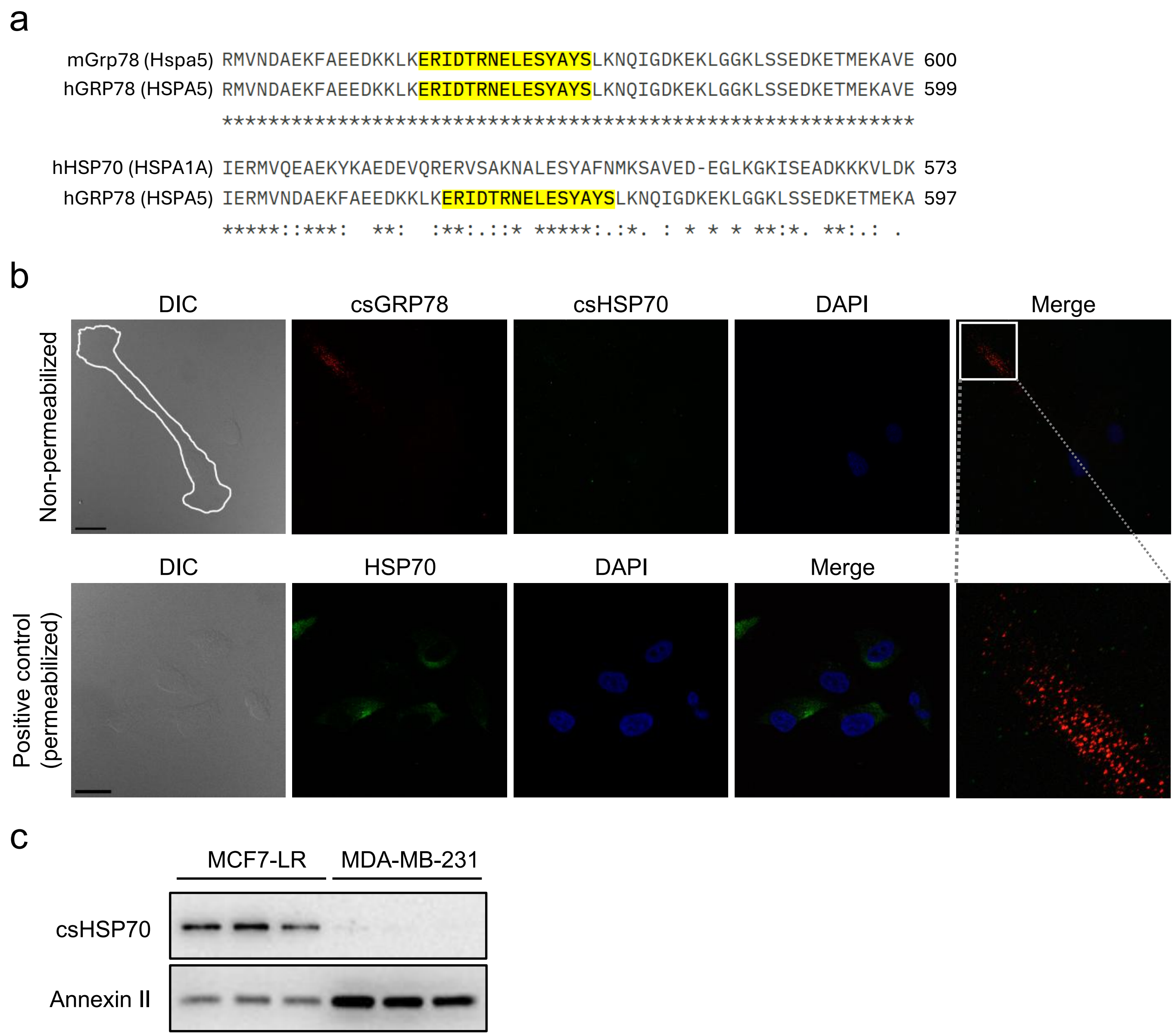

Fig. S3 MDA-MB-231 cells exhibit minimal cell-surface HSP70 detectable only by biochemical analysis and not by immunofluorescence. a Multiple sequence alignment highlights the epitope of the 76-E6 antibody in mouse Grp78, human GRP78, and human HSP70 proteins. **b** Upper panels: Representative immunofluorescence and single optical section of confocal images at the thickness of 0.3 μm showing surface GRP78 (red; MAb159) and HSP70 (green; C92F3A-5) on non-permeabilized MDA-MB-231 cells (n=13 from 8 image areas); nucleus stained with DAPI (blue). The cell periphery was outlined with the white line in the DIC image. The boxed area was enlarged below. Lower panels: MDA-MB-231 cells were permeabilized with 0.1% Triton X-100, followed by staining with anti-HSP70 antibody (green; C92F3A-5); nuclei stained with DAPI (blue). Scale bars, 20 μm. **c** Western blot analysis was performed with biotinylated cell surface proteins extracted from tamoxifen-resistant MCF7-LR breast cancer cells and MDA-MB-231 cells. The C92F3A-5 anti-HSP70 antibody was used in this study. Annexin II is a loading control. This experiment was performed with 3 biological samples per cell line. The full-length images are available in Fig. S10 of Supplementary Information 2.

CD44v

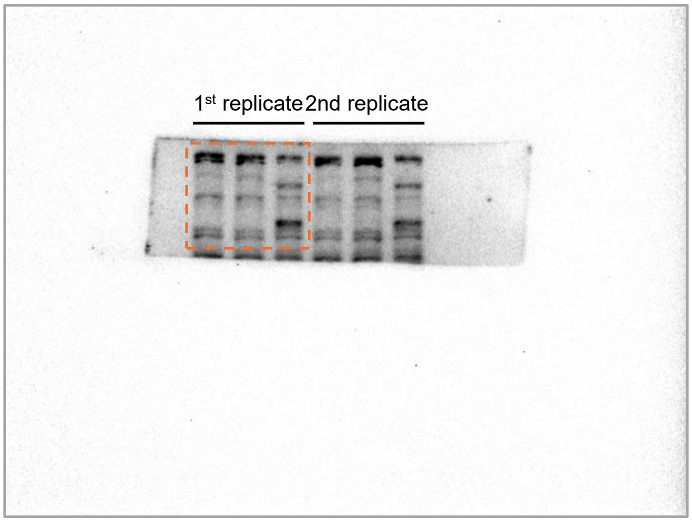

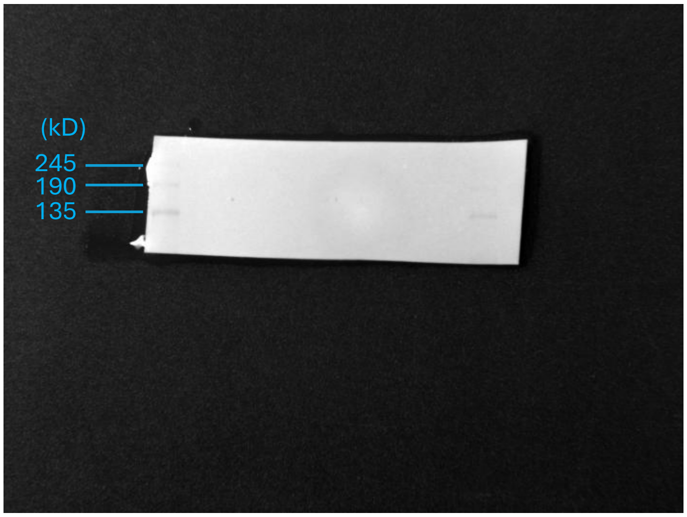

Protein ladder: NEB #P7712

GAPDH (shorter exposure)

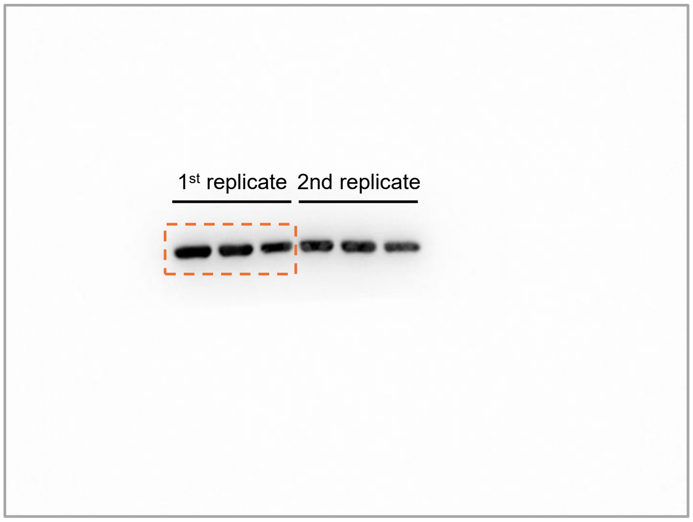

GAPDH (longer exposure)

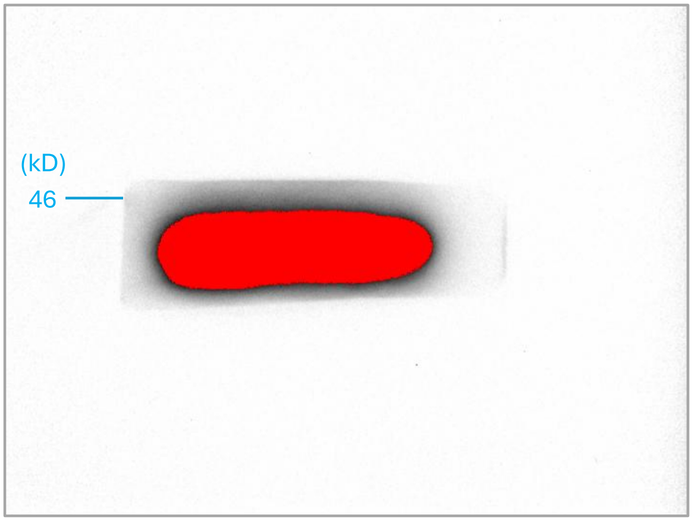

Fig. S4 The full-length images for Figure 1f (first gel of the experiment).

pSrc(Y419)

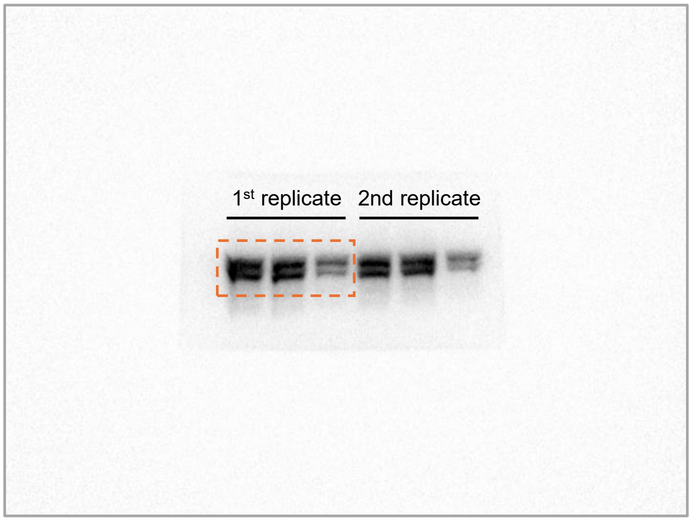

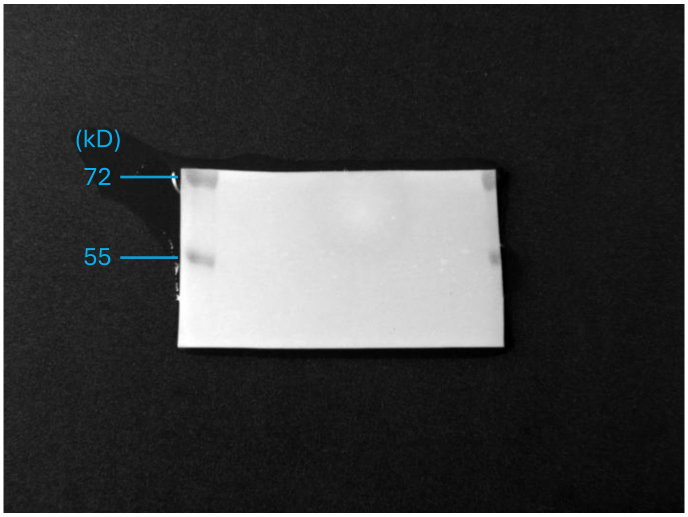

Protein ladder: NEB #P7712

tSrc

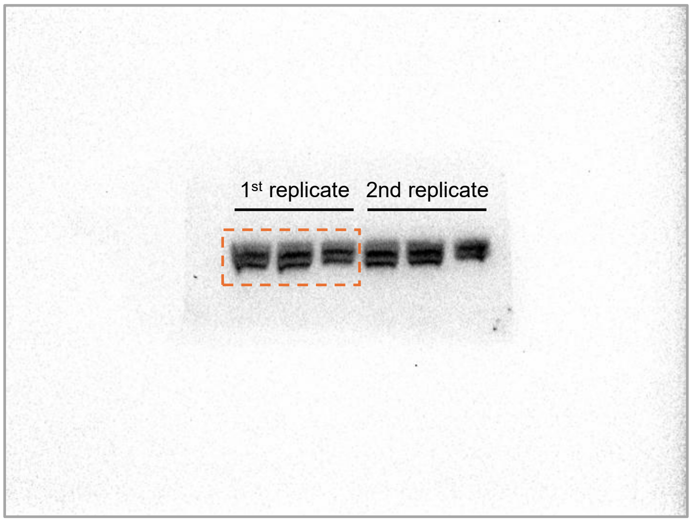

Fig. S5 The full-length images for Figure 1f (second gel of the experiment).

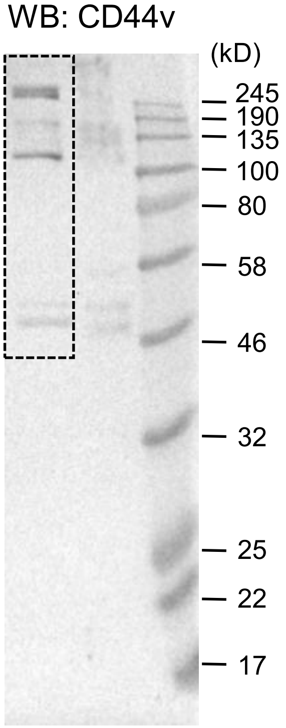

Fig. S6 The full-length image for Figure S1a.

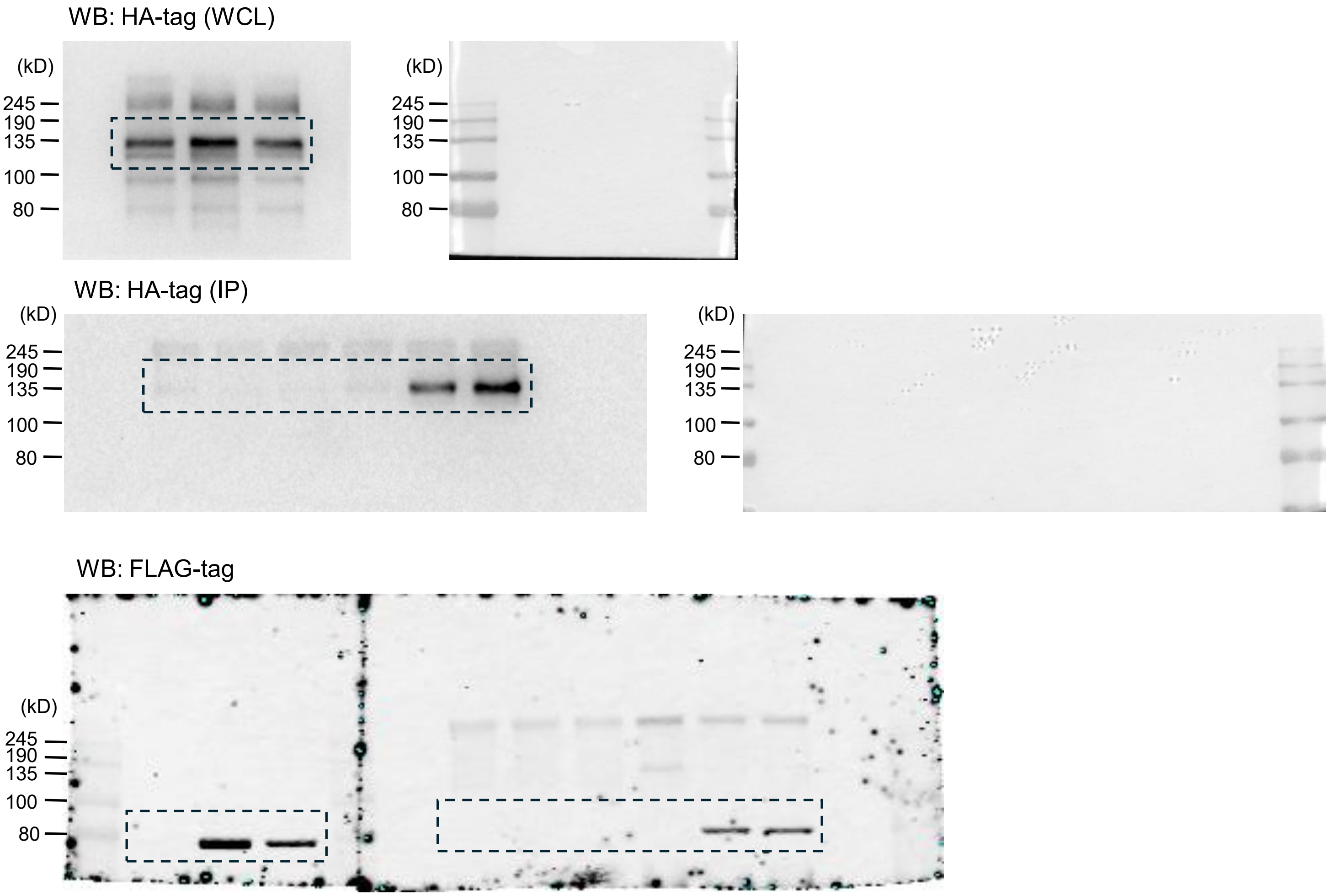

Fig. S7 The full-length image for Figure S1c.

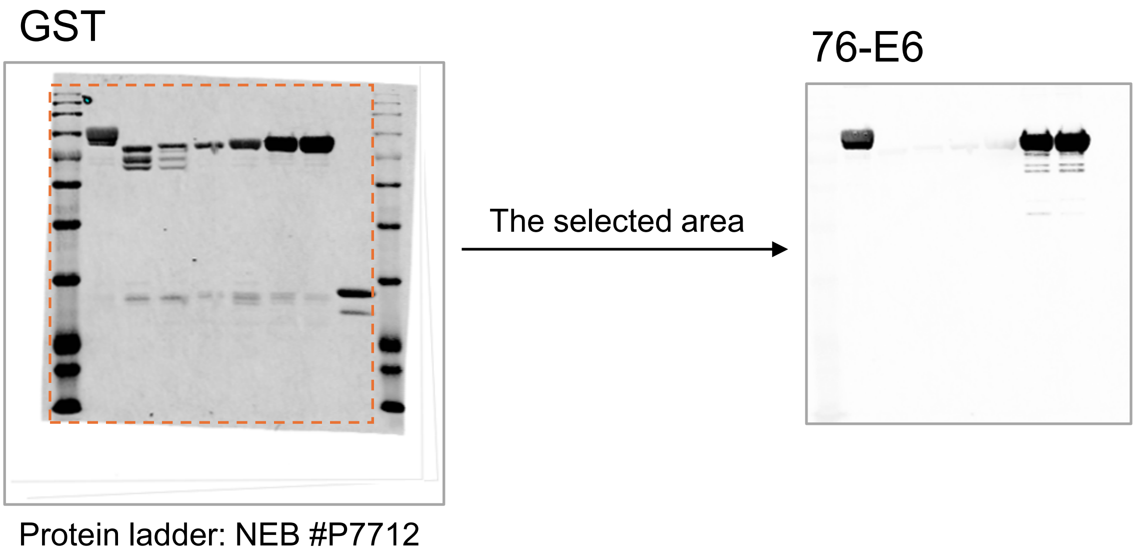

Fig. S8 The full-length images for Figure 3a.

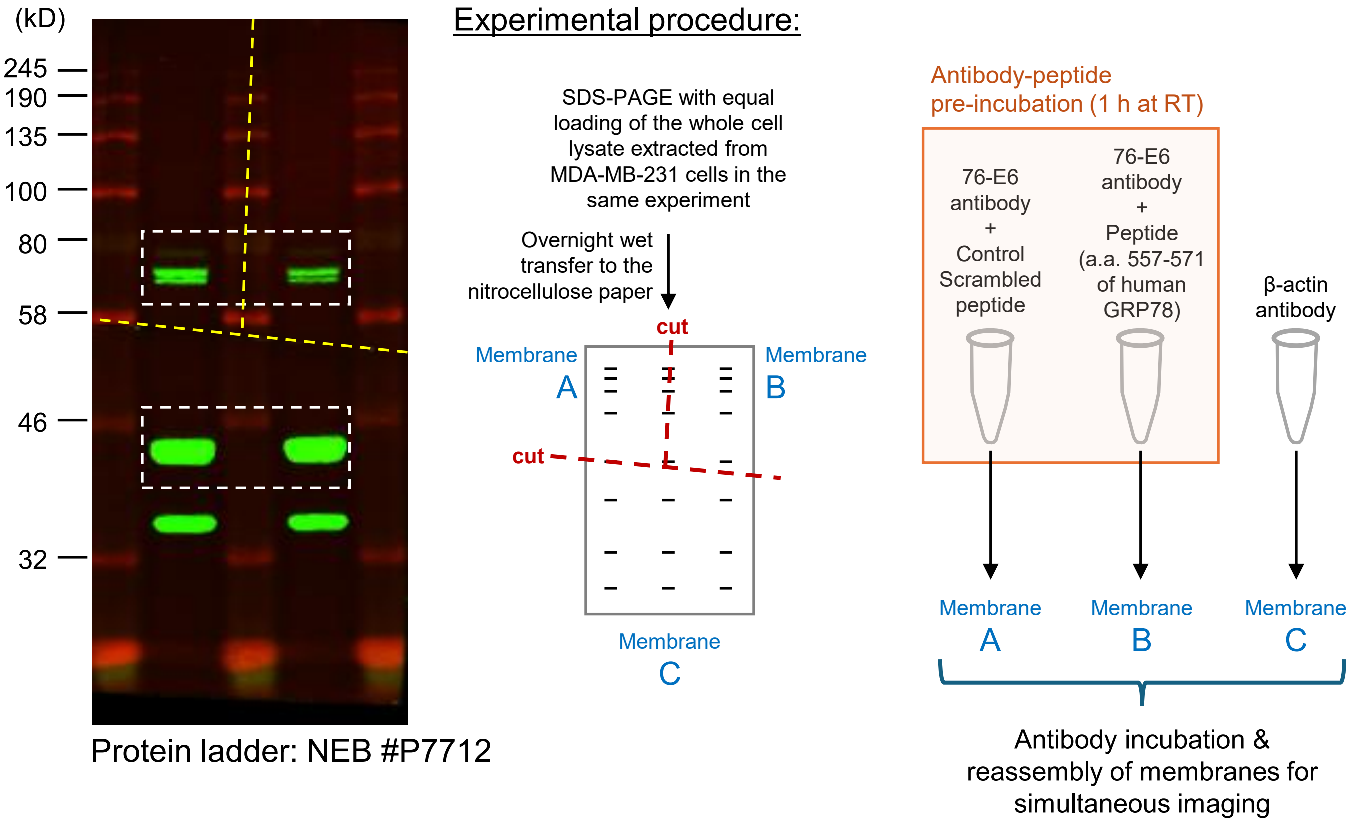

Fig. S9 The full-length image and experimental procedure for Figure 3b.

csHSP70

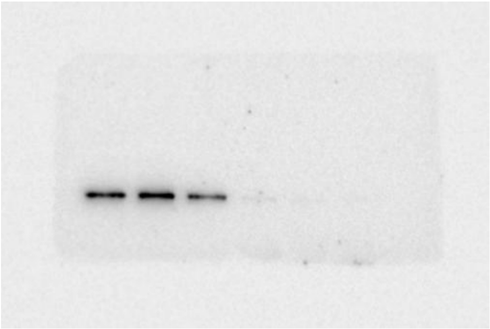

Annexin II

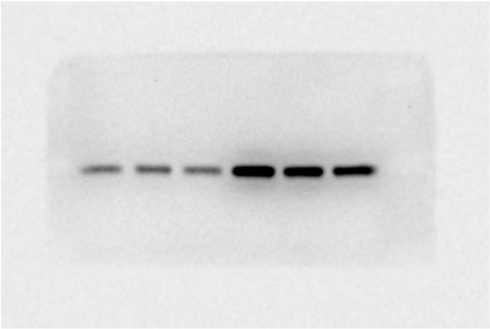

Fig. S10 The full-length image for Figure S3c.
